# Internet and Mobile Technology Use Among Urban African American Parents: Survey Study of a Clinical Population

**DOI:** 10.2196/jmir.2673

**Published:** 2014-01-13

**Authors:** Stephanie J Mitchell, Leandra Godoy, Kanya Shabazz, Ivor B Horn

**Affiliations:** ^1^Children's National Medical CenterCenter for Translational ScienceGeorge Washington University School of MedicineWashington, DCUnited States; ^2^Children's National Medical CenterDivision of General Pediatrics/Center for Translational ScienceGeorge Washington University School of MedicineWashington, DCUnited States

**Keywords:** health communication, mobile phone, social networking, African Americans, pediatrics

## Abstract

**Background:**

There is considerable potential for mobile technologies to empower pediatric patients and families by improving their communication with health professionals. National surveys suggest minority parents frequently communicate via mobile technology, but it is uncertain how amenable they are to receiving health care information in this format. Although the low cost and far reach characteristics of mobile health (mHealth) technology makes it advantageous for communication with minority parents, data on acceptance are needed.

**Objective:**

The objective of the study was to determine utilization of mobile and Internet technology by African American parents in an urban, underserved population, and to assess their interest in receiving health information via text messaging or other technologies (eg, social media and the Internet).

**Methods:**

A survey was administered to parents of children aged 1-12 years covered by public insurance receiving care at 3 pediatric primary care centers in Washington, DC.

**Results:**

The African American sample (N=302) was composed of primarily single (75.8%, 229/302) mothers. Almost half had more than a high school education (47.7%, 144/302) and incomes above US $25,000 per year (43.0%, 130/302). Most (97.0%, 293/302) reported owning a cell phone, of which 91.1% (275/302) used it to text and 78.5% (237/302) used it to access the Internet. Most had service plans with unlimited text and data, but 26.5% (80/302) experienced service interruptions in the previous year. Home Internet access was more prevalent among those with higher income (86.2%, 112/130), but it was still relatively pervasive among lower income families (66.9%, 83/124). In adjusted logistic regression models, African American mothers with income greater than US $25,000 annually were 4 times as likely to own a tablet computer than their lower income counterparts. Of the participants, 80.8% (244/302) used social networking, primarily Facebook, and 74.2% (224/302) were interested in joining a social networking group about a health topic concerning their child. Although relatively few African American mothers (17.9%, 54/302) shared health information via texting, there was strong interest in receiving health information via mobile phones (87.4%, 264/302). There was no significant difference in Internet/mobile device use or interest in using these outlets to send/receive information about their children’s health between parents of healthy children and parents of children with chronic health conditions.

**Conclusions:**

Urban African American parents are active users of the Internet and mobile technology for social interactions, but they are less likely to use it for accessing or communicating health information. However, most parents expressed an interest in receiving health information or utilizing social networking to learn more about health topics. Mobile technology and social networks may be an underutilized method of providing health information to underserved minority populations.

## Introduction

As with all forms of Web-based technology, advances in mobile health (mHealth) and health communication technology are rapidly increasing. According to the National Institutes of Health Consensus Group, mHealth is defined as the use of mobile and wireless devices to improve health outcomes, health care services, and health research [1]. Health communication technology is used specifically for communication between patients or caregivers and health care providers about health [2], and it is typically a means of streamlining the delivery of health information and services [3]. Some of the many advantages of mHealth communication technologies include the relatively low cost and the ease with which apps and text messages can be widely distributed because of the popularity of mobile technology.

Data from the Pew Research Center’s Internet & American Life Project indicate that technology use has expanded rapidly over the past decade and is now pervasive [4,5]. As of spring 2013, approximately 85% of American adults used the Internet, 56% owned a smartphone, and 63% went online wirelessly with a laptop or cell phone [4-6]. Furthermore, 72% of Internet users reported looking online for health information; 31% of cell phone owners and 52% of smartphone owners say they have used their phone to look up health or medical information [7]. Despite overall increases in access to and use of the Internet and mHealth technology, differences in access and use vary by race/ethnicity, age, income, and education [4,8]. With regard to race and ethnicity, current (2013) Internet usage rates for non-Hispanic black adults (85%) are comparable with those of non-Hispanic white adults (86%), with usage rates for Hispanic adults lagging slightly behind (76%) [5]. However, underrepresented minority (URM) populations, including African Americans and English-speaking Hispanic persons, are more likely to own a mobile phone and to use mobile technology than their white counterparts [9]. Moreover, Hispanic persons and African Americans are more likely than white cell phone users to look for health information on their phones [10].

At the same time, URMs experience higher rates of childhood chronic diseases. The highest prevalence of asthma in the United States is among non-Hispanic black children who are almost 7 times more likely to die of asthma than white children [11]. URM children are also at high risk for obesity and type 2 diabetes [12]. Because of its popularity among URM populations, its relatively low cost, and its demonstrated efficacy in facilitating parent-provider communication and improving health behaviors, mHealth communication technology is uniquely well-suited for addressing pediatric health disparities. mHealth technology has been successfully used to improve patient-provider communication [2,13], chronic disease self-management [14], and preventive health behaviors [15]. The efficacy of mHealth technologies is, in part, because of their convenience as a method of health information exchange [16] and they are effective for modifying health behavior because behavioral cues (eg, reminders) can be sent/received asynchronously when and where they are most relevant or appropriate [17-18].

One arena in which mHealth communication technology can have important public health effects is in pediatric settings, where clear and frequent communication between parents and providers is important for managing children’s chronic diseases (eg, asthma, diabetes). Parents (or other caregivers) serve as the intermediary between pediatric patients and providers; they determine when health care is sought and are primarily responsible for relaying information about their child’s health to providers [2]. Thus, facilitating parent-provider communication through mobile technology is a promising method for addressing disparities in childhood chronic diseases.

Although there is great potential for mHealth to reduce pediatric health disparities, more information is needed to understand how URM parents/patients use communication technology, particularly in reference to their children’s health-related issues. Most of the research among African American parents has focused on their use of and access to Web-based health information. Not only do the reported rates of Internet use vary by population sampled, but these data are out of date considering how rapidly and pervasively Internet-capable mobile devices are being disseminated [8,19-20]. More specific information is needed on URM parents’ usage patterns and receptivity to receiving health information via mobile technology. To our knowledge, only 1 study has been conducted on urban parents’ use of mobile technology within a clinical pediatric setting [21]. This study affirmed the widespread popularity and frequent use of mobile technology (eg, 75% of respondents used some form of digital technology daily), including searching for health information (eg, 58% searched for medical info at least once in the preceding week) among families seeking care from urban pediatric primary care centers. However, information was not gathered in several areas that would be necessary to understand the way in which mobile technology could be used to address health disparities, such as participants’ race/ethnicity, the impact of child health status on use, service interruptions, functions of cell phone use, and specific modes in which parents may be interested in accessing health information.

The current study reports on the use of mobile phones, the Internet and wireless devices, and social media among a sample of urban minority parents of children attending pediatric community health clinics. To help guide the development and marketing of mHealth communication technologies for URM parents, we examine how patterns of access, use, and preferences relating to mHealth technology vary by socioeconomic characteristics (ie, education, income). We additionally explore the extent to which parents of children with chronic diseases differ from other parents in their use of technologies due to the particular potential of mHealth in addressing childhood chronic conditions.

## Methods

### Participants

A clinical sample of parents whose children received primary care services at 1 of 3 pediatric primary care practices in the Washington, DC metropolitan area between July 2011 and December 2012 were recruited to participate in the study. A clinical sample, as opposed to a sample drawn from the general population of minority parents of young children, was chosen to understand how providers may better engage and communicate with parents through technology. English-speaking parents were eligible if they were at least 18 years old, their child’s legal guardian, had a child aged between 12 months and 12 years, and they could identify a primary care provider for the child. For this analysis, the sample (N=314) was constrained to self-identified African American parents (n=302).

### Procedures

Trained research assistants invited parents/guardians to participate while they were in the pediatric clinic waiting area, and screened interested parents for eligibility. Upon meeting inclusion criteria and providing informed consent, parents/guardians completed the 5-10 minute paper survey. The Children’s National Health System Institutional Review Board approved all study procedures.

### Measures

A demographics questionnaire was administered to collect information on parent age, race/ethnicity (self-reported by the parents of the children from a list including white/Caucasian, black/African American, Asian/Pacific Islander, American Indian/Alaskan, Latino/Hispanic, or other), marital status, relationship to child, household composition, annual income, education level, occupation, and child’s health status (ie, diagnosis of chronic illness). Parents indicated the level of education they had completed from the following choices: some grade school, completed junior high school, high school graduate or graduate equivalency degree (GED), some college/2-year college, college graduate, and postgraduate study. Based on the distribution, responses were recoded into less than a high school degree/GED or more than a high school degree/GED. Similarly, parents’ selection from 6 categories of annual household income (< US $10,000, US $10,000-US $24,999, US $25,000-US $49,999, US $50,000-US $74,999, US $75,000-US $99,999, and ≥US $100,000) were recoded into < US $25,000 and ≥ US $25,000.

The survey measure of mobile and Internet technology utilization was an adaptation of the Pew Internet & American Life Project, a telephone survey of 2252 Americans older than 18 years [4]. The Pew survey included 23 multiple-choice questions covering topics such as ownership of electronic devices, Internet access and usage, mobile phone plans, and utilization of mobile phone features, such as mobile Internet and email, text messages, video messages, etc. Our adapted measure followed a similar format with 27 additional questions, including 14 regarding mobile phone use, 9 regarding Internet usage, and 4 regarding social networking use.

### Statistical Analyses

Frequencies were generated to characterize cell phone, Internet, and social networking use as well as parents’ interest in receiving child health information through these channels. Next, chi-square tests were conducted comparing frequencies/proportions of each technology use outcome across groups defined by education (> high school vs ≤high school), annual income (>US $25,000 vs ≤US $25,000), and health status of children (chronic illness vs no chronic illness).

Any socioeconomic (eg, education, income) differences that were significant according to chi-square tests were further examined using a logistic regression adjusting for the other predictor variables. Statistical significance was set at *P*=.01 because of the number of tests conducted and the higher chances of type I (false positive) errors.

## Results

### Sample Demographics

See Table 1 for participant characteristics. This sample included primarily single (75.8%, 229/302) mothers (84.1%, 254/302) with an average of 2.5 (SD 1.6) children. Participants ranged in age from 18 to 59 years (mean 31.5, SD 8.0), and household sizes ranged from 1 to 10 persons (mean 4.2, SD 1.7). Close to half had more than a high school education (47.7%, 144/302) and incomes greater than US $25,000 per year (43.0%, 130/302). Almost one-third had a child with asthma (31.1%, 94/302); 53.6% (162/302) had children without any chronic illness.

**Table 1:** Demographic characteristics of African American participants (N=302).

Demographics		Mean (SD)	Range	n (%)
Parent age (years)		31.5 (8.0)	18-59	
**Relationship to child**				
	Biological mother			254 (84.1)
	Biological father			26 (8.4)
	Legal guardian			21 (7.0)
**Marital status**				
	Married			51 (16.9)
	Single			229 (75.8)
	Divorced/separated			18 (6.0)
Number of children		2.5 (1.6)	1-10	
**Household total**		4.2 (1.7)	1-10	
	Children	2.4 (1.4)	0-7	
	Adults	1.9 (0.9)	1-7	
**Highest education**				
	Less than high school			18 (6.0)
	High school/GED			138 (45.7)
	Some college			110 (36.4)
	Bachelor degree or more			34 (11.3)
**Income (US $)**				
	<10,000			58 (19.2)
	10,000-24,999			66 (21.9)
	25,000-49,999			87 (28.8)
	50,000-74,999			25 (8.3)
	75,000-99,999			11 (3.6)
	>100,000			7 (2.3)
	Don’t know			40 (13.2)
**Child chronic health problems**				
	None			162 (53.6)
	Asthma			94 (31.1)
	Diabetes			2 (0.7)
	Obesity			2 (0.7)
	Other			14 (4.6)
	Multiple conditions			14 (7.3)

### Cell Phone Use

When asked “what do you use your cell phone for,” most participants selected functions in addition to voice calls: 91.1% (275/302) text, 70.9% (214/302) send/receive email, and 78.5% (237/302) access the Internet (Table 2). Most had service plans with unlimited texts (86.4%, 261/302) and data (76.5%, 231/302), but 26.5% (80/302) experienced service interruptions (of unknown duration) in the previous year. When asked to indicate how many texts they sent/received on an average day, 18.2% (55/302) said they sent/received more than 50. Although most (66.2%, 200/302) sent messages about important personal matters, only 17.9% (54/302) reported sharing health information via text.

**Table 2:** Participant cell phone use by education and income (N=302).

Cell phone use		Overall n (%)	Parent education n (%)		Household income (US $) n (%)	
			≤High school (n=156)	>High school (n=144)	<25,000 (n=124)	≥25,000 (n=130)
Do you have a cell phone? (yes)^a^		293 (97.0)	94.2 (147)	144 (100)	118 (95.2)	127 (97.7)
**What do you use your cell phone for?**						
	Calls^a^	288 (95.4)	143 (91.7)	144 (100)	116 (93.5)	127 (97.7)
	Texts^a^	275 (91.1)	135 (86.5)	139 (96.5)	112 (90.3)	120 (92.3)
	Emails^a^	214 (70.9)	102 (65.4)	112 (77.8)	83 (66.9)	99 (76.2)
	Internet	237 (78.5)	116 (74.4)	120 (83.3)	98 (79.0)	103 (79.2)
**Service plan**						
	My cell phone plan includes text messaging with...unlimited texts	261 (86.4)	128 (89.5)	131 (91.0)	106 (90.6)	116 (91.3)
	My plan includes data with...unlimited data	231 (76.5)	113 (77.9)	116 (81.7)	94 (79.7)	104 (83.2)
	In the past year, has your cell phone service been interrupted? (yes)	80 (26.5)	43 (29.5)	36 (25.0)	39 (33.1)	36 (28.3)
**Who do you text?**						
	Family^a^	273 (90.4)	132 (84.6)	139 (96.5)	111 (89.5)	122 (93.8)
	Friends	278 (92.1)	139 (89.1)	137 (95.1)	113 (91.1)	122 (93.8)
	Coworkers^a,b^	135 (44.7)	53 (34.0)	82 (56.9)	46 (37.1)	81 (62.3)
**On an average day, how many text messages do you send or receive on your cell phone?**						
	≤10^a^	55 (18.2)	21 (14.6)	33 (23.6)	19 (16.4)	29 (23.6)
	11-25^a^	87 (28.8)	35 (24.3)	51 (36.4)	34 (29.3)	45 (36.6)
	26-50^a^	63 (20.9)	37 (25.7)	51 (18.6)	28 (24.1)	24 (19.5)
	>50	55 (18.2)	30 (20.8)	25 (17.9)	26 (22.4)	20 (16.3)
**When you text do you...?**						
	Just say hello and chat^a,b^	248 (82.1)	119 (76.3)	127 (88.2)	101 (81.5)	11 (85.4)
	Do things related to work	127 (42.1)	52 (33.3)	75 (52.1)	49 (39.5)	71 (54.6)
	Send multiple messages to discuss important personal matters^a^	200 (66.2)	93 (59.6)	106 (73.6)	78 (62.9)	97 (74.6)
	Share information about your health	54 (17.9)	29 (18.6)	25 (17.4)	30 (24.2)	16 (12.3)

^a^Chi-square tests indicate a significant (*P*<.01) difference between parents with ≤ high school vs > high school education.

^b^Chi-square tests indicate a significant (*P*<.01) difference between parents with annual household incomes < US $25,000 vs ≥ US $25,000.

There were several significant socioeconomic differences in terms of cell phone use. Chi-square tests (Table 2) indicated that parents with more than a high school education were more likely to own a cell phone (χ^2^_1_=8.7, *P*=.003) and use nonvoice phone features (eg, text messaging; χ^2^_1_ = 9.7, *P*=.002). Multivariate logistic regression (Table 3) controlling for household income also showed that parents with more than a high school education were 6 times more likely to use their cell phones to send text messages compared to parents with less education (*P*=.002).

Chi-square tests suggested that those with higher incomes (>US $25,000) were more likely to use cell phones for work purposes (eg, to text coworkers: χ^2^_1_ =17.2, *P*<.001; send messages related to work: χ2_1_ =6.0 *P*=.02) than those with lower incomes (Table 2). The income-related difference in likelihood of texting coworkers remained significant in the multivariate model (Table 3) controlling for parent education (*P*=.001). There were no significant differences in cell phone use by child health status (results not shown).

**Table 3:** Odds of reporting various cell phone, Internet, and social network usage for parents with more than a high school education or a household income above US $25,000.^a^

Technology use			>High school education		Household income ≥ US $25,000	
			OR	95% CI	OR	95% CI
**Cell phone use**						
	Own cell phone		0	0	1.04	0.25-4.40
	**Uses these functions:**					
		Calls	0	0	1.43	0.36-5.70
		Texts	6.23^b^	1.96-19.83	0.76	0.30-1.95
		Emails	1.78	0.99-3.21	1.28	0.71-2.30
	**Sends texts to these people:**					
		Family	5.12^b^	1.60-16.38	1.13	0.43-2.97
		Coworkers	1.58	0.92-2.69	2.41^b^	1.41-4.11
	Send/receive >50 texts/day on average		0.89^b^	0.45-1.75	0.71	0.36-1.39
	**When you text do you...?**					
		Just say hello and chat	2.26	1.09-4.66	1.04	0.51-2.10
		Do things related to work	1.53	0.87-2.69	1.54	0.87-2.71
		Send multiple messages to discuss important personal matters	1.47	0.84-2.58	1.49	0.85-2.61
		Share information about your health	0.92	0.47-1.82	0.45	0.22-0.90
**Internet usage**						
	**Do you own any of the following items?**					
		None	0.34^b^	0.15-0.76	0.37^b^	0.17-0.82
		Desktop computer	1.61	0.94-2.75	2.44^b^	1.43-4.17
		Laptop or notebook	1.70	0.99-2.92	2.11^b^	1.22-3.62
		iPod or MP3 player	1.60	0.94-2.73	2.18^b^	1.28-3.72
		Game console	1.02	0.60-1.74	2.10^b^	1.23-3.57
		Tablet computer	1.12	0.45-2.76	4.14^b^	1.44-11.89
	Have Internet access at home		1.68	0.89-3.17	2.65^b^	1.38-5.09
	**Ever used Internet for...**					
		Send/read email	5.24^b^	2.08-13.17	0.74	0.33-1.66
		Get news online	3.29^b^	1.75-6.18	1.23	0.67-2.27
		Get health info	1.98	1.16-3.37	0.88	0.52-1.51
**Social networking (which sites)**						
		MySpace	1.03	0.41-2.58	0.23^b^	0.08-0.67
		LinkedIn	5.10	1.09-23.79	2.91	0.78-10.91

^a^Models include both greater than high school education and household income above US $25,000 so that ORs reflect independent influence of each predictor while adjusting for the other.

^b^OR is significant (*P*<.01).

### Internet Use

In this sample, most parents owned either a desktop (43.7%, 132/302) or laptop computer (55.3%, 167/302), and approximately three-quarters (75.5%, 228/302) reported having Internet access at home (see Table 4). Most parents (69.5%, 210/302) reported using the Internet to get news or visit social networking sites (69.9%, 211/302), whereas only 53.0% (160/302) reported using the Internet to get health information. Nevertheless, more than 87% (264/302) reported willingness or interest in receiving health info online through email or in texts.

Chi-square analyses (Table 4) indicated that parents with more education were more likely to own a computer (χ^2^_1_ = 18.6, *P*<.001) and have Internet access at home (χ^2^_1_ = 10.4, *P*=.001). In addition, those with more education were more likely to use the Internet to send/read email (χ^2^_1_ = 22.9, *P*<.001), get news (χ^2^_1_ = 23.5, *P*<.001), and get health information (χ^2^_1_ = 9.7, *P*=.002) than those with less than a high school education. When controlling for household income (Table 3), parents with more education were more likely to own an Internet-capable device (*P*=.009) and to use the Internet for email and news (*P*<.001). Those with more than a high school education were more likely to seek health information online than those with less education, but this did not reach statistical significance (*P*=.03).

Chi-square tests (Table 4) and multivariate logistic regressions (Table 3) revealed that parents with higher incomes were more likely to own all types of Internet-capable devices (χ^2^_1_ = 12.1, *P*=.001) and twice as likely to have Internet access at home (*P*=.002). There were no significant differences in Internet use or interest in online health information by child health status (results not shown).

**Table 4:** Participant Internet use by education and income (N=302).

Internet use		Overall n (%)	Parent education n (%)		Household income (US$) n (%)	
			≤High school (n=156)	>High school (n=144)	<25,000 (n=124)	≥25,000 (n=130)
**Do you own any of the following items?**						
	None^a,b^	52 (17.2)	41 (26.3)	11 (7.6)	29 (23.4)	10 (7.7 )
	Desktop computer^a,b^	132 (43.7)	53 (34.0)	78 (54.2)	41 (33.1)	76 (58.5)
	Laptop or notebook^a,b^	167 (55.3)	70 (44.9)	96 (66.7)	57 (46.0)	93 (71.5)
	iPod or MP3 player^a,b^	114 (37.7)	46 (29.5)	68 (47.2)	37 (29.8)	67 (51.5)
	Game console^b^	150 (49.7)	71 (45.5)	79 (54.9)	53 (42.7)	80 (61.5)
	Tablet computer^b^	26 (8.6)	9 (5.8)	17 (11.8)	5 (4.0)	20 (15.4)
Do you have Internet access at home (other than a cell phone)? (yes)^a,b^		228 (75.5)	106 (67.9)	120 (83.3)	83 (66.9)	
**Have you ever used the Internet to do any of the following?**						
	Send/read email^a^	254 (84.1)	116 (74.4)	136 (94.4)	107 (86.3)	115 (88.5)
	Get news online^a^	210 (69.5)	90 (57.7)	119 (82.6)	85 (68.5)	103 (79.2)
	Online chat	155 (51.3)	79 (50.6)	76 (52.8)	69 (55.6)	64 (49.2)
	Social networking site	211 (69.9)	102 (65.4)	108 (75.0)	89 (71.8)	93 (71.5)
	Write or read blogs	161 (53.3)	78 (50.0)	82 (56.9)	61 (49.2)	74 (56.9)
	Use Twitter	102 (33.8)	50 (32.1)	51 (35.4)	41 (33.1)	46 (35.4)
	Watch video sharing site	191 (63.2)	92 (59.0)	98 (68.1)	81 (65.3)	83 (63.8)
	Search engine	209 (69.2)	101 (64.7)	107 (74.3)	91 (73.4)	89 (68.5)
	Get health info^a^	160 (53.0)	68 (43.6)	91 (63.2)	67 (54.0)	73 (56.2)
	Video games	121 (40.1)	59 (37.8)	61 (42.4)	56 (45.2)	51 (39.2)
Would you be willing to receive email or text messages to get health information? (yes)		264 (87.4)	141 (91.0)	121 (84.0)	112 (90.3)	109 (83.8)
Would you be Interested in receiving health information on the Internet via email or online? (yes)		255 (84.4)	133 (85.8)	120 (83.9)	109 (88.6)	109 (83.8)
Would you be interested in keeping track of your child’s health online? (yes)		279 (92.4)	141 (91.0)	136 (94.4)	115 (92.7)	120 (92.3)

^a^Chi-square tests indicate a significant (*P*<.01) difference between parents with ≤high school vs >high school education.

^b^Chi-square tests indicate a significant (*P*<.01) difference between parents with annual household incomes <US $25,000 vs ≥US $25,000.

### Social Networking

More than 80.8% (244/302) of parents in our sample used social networking, primarily Facebook (78.8%, 238/302), and close to half (46.4%, 140/302) accessed these sites daily (see Table 5). Almost three-quarters (74.2%, 224/302) were interested in joining a social networking group about a health topic concerning their child.

There were very few education or income differences in social networking activity among African American mothers. Chi-square tests (χ^2^_1_ = 9.8, *P*=.002) and multivariate logistic regressions (Table 3; *P*=.005) indicated that those with lower incomes were more likely to use MySpace. Also, chi-square tests (Table 5) showed parents with more education (χ^2^_1_ = 11.4, *P*=.001) and higher household incomes (χ^2^_1_ = 7.0, *P*=.008) were more likely to use LinkedIn compared to their counterparts, but multivariate results suggest the effects of education and income were not independent (Table 3). There were no significant differences in social networking by child health status (results not shown).

**Table 5:** Participant social networking by education and income (N=302).

Social networking use		Overall n (%)	Parent education n (%)		Household income (US $) n (%)	
			≤High school (n=156)	>High school (n=144)	<25,000 n=124)	≥25,000 (n=130)
Ever use social networking		244 (80.8)	130 (83.3)	112 (77.8)	104 (83.9)	99 (76.2)
**Which social networking sites do you use?**						
	Facebook	238 (78.8)	126 (80.8)	110 (76.4)	102 (82.3)	96 (73.8)
	MySpaceb	24 (7.9)	14 (9.0)	10 (6.9 )	18 (14.5)	5 (3.8)
	LinkedIn^a,b^	16 (5.3)	2 (1.3)	14 (9.7 )	3 (2.4)	13 (10.0)
	Twitter	90 (29.8)	47 (30.1)	41 (28.5)	41 (33.1)	35 (26.9)
**How often do you visit social networking sites?**						
	Every day	140 (46.4)	74 (57.4)	65 (58.0)	55 (52.9)	61 (61.6)
	2-3 days/week	50 (16.6)	25 (19.4)	25 (22.3)	24 (23.1)	16 (16.2)
	Weekly	33 (10.9)	18 (14.0)	14 (12.5)	17 (16.3)	12 (12.1)
	Monthly	20 (6.6)	12 (9.3)	8 (7.1 )	8 (7.7)	10 (10.1)
Would you join a social networking group about a health topic concerning your child? (yes)		224 (74.2)	114 (74.5)	108 (78.8)	98 (83.1)	94 (74.0)

^a^Chi-square tests indicate a significant (*P*<.01) difference between parents with ≤high school vs >high school education.

^b^Chi-square tests indicate a significant (*P*<.01) difference between parents with annual household incomes <US $25,000 vs ≥US $25,000.

## Discussion

This study aimed to guide the development of mHealth communication technologies designed for URM pediatric patients, particularly technologies that could address chronic conditions for which there are racial/ethnic disparities. We examined patterns of mHealth communication technology access, use, and preferences among a socioeconomically diverse sample of African American parents of underserved children in urban community health centers.

Our data confirm that African American adults, primarily mothers in this case, are avid users of mobile phones. Nearly everyone in our sample (97%) reported owning a cell phone, which is similar to recent findings by the Pew Research Center on African American cell phone ownership (93%) [9]. Approximately 90% of all participants used their cell phones to text, and approximately 80% used them to access the Internet. These rates are even higher than those for non-Hispanic black adults reported in Pew’s May 2012 national survey (80% and 60% for texting and Internet access, respectively) [22]. The higher rates in our sample may be due to participants’ residing in a metropolitan area [23], but they also may be related to the participants’ specific age and status as parents which has an associated need to be easily accessible. The health communication implication of their pervasive use of mobile phones for texting and Internet access is that these channels may be a more acceptable means than voice calls for health care providers to communicate with parents/caregivers of pediatric patients. Although relatively few African American mothers (18%) shared health information via text, there was strong interest in receiving health information via mobile phones (87%). Thus, findings support both high levels of nonvoice mobile phone use and high levels of interest in receiving health information via this channel.

A caveat in the ubiquity of mobile phone use is that over 25% of African American parents in our sample experienced cell phone service interruption in the prior year. This means that for a notable proportion of African American mothers, mobile phones are not a totally reliable form of communication. Nevertheless, there is probably not a more consistent means of contacting these parents/caregivers. We know of no other studies that have examined the frequency and pervasiveness of cell phone service interruptions. Therefore, it would be useful to collect more information about service interruptions and turnover in phone numbers when designing mHeath interventions for this population.

Our data also indicate that most urban African American mothers can access the Internet at home (76%). Although home Internet access in our sample was more prevalent among those with higher income (86%), it was still relatively pervasive among lower income families (67%). Our findings regarding home Internet access were similar to those of DeMartini and colleagues [21], who found that 80% of their sample of urban parents had home Internet access. Also, our finding that lower income parents were less likely to report home Internet access parallels the Pew Center data, although national rates for adults are lower (46% of those with annual incomes less than US $30,000 reported home broadband access) [24].

In addition to discrepancies in home Internet access, there were also significant socioeconomic discrepancies in mobile device ownership. Although there were no significant differences in cell phone ownership, mothers with higher incomes were at least twice as likely to own various other mobile devices (eg, laptop, tablet computer) than their lower income counterparts, which parallels the Pew Center’s 2013 survey data [25]. This income disparity is important for developers to account for when considering the platforms and user interface of apps and other mHealth technologies.

We found that email and websites, including social networks, are other venues with large potential for communicating with URM parents about children’s health management because 84% reported email use and 70% reported social networking use (46% reported daily use). Our findings regarding the frequency of email and social networking use parallel data on all adults from the Pew Center (88% of Internet users send or receive email and 67% use a social networking site) [26] and from DeMartini and colleagues (91% of parents reported having an email account) [21]. Moreover, 84% of our sample reported interest in receiving health information on the Internet via email or online, and more than half are already using the Internet to get health information. Despite potential privacy concerns, 74% of mothers were interested in joining a social networking group about a health topic concerning their children. DeMartini and colleagues similarly found among their sample of urban parents, strong interest in the use of mHealth data with more than 70% of parents reporting that they would use digitally supplied health information.

We found no significant difference in Internet/mobile device use or interest in using these outlets to send/receive information about their children’s health between parents of healthy children and parents of children with a chronic health condition. We know of no other study that has examined this issue, and more research is needed to confirm our findings. Yet, our results suggest that mHealth could be harnessed to reduce racial disparities across both chronic diseases and more general child health measures (eg, vaccination receipt)[27].

This survey of urban African American mothers of pediatric primary care patients is an important first step in understanding acceptability of mHealth communication technology in this population, but there is more specific and nuanced information that will be necessary for designing technologies targeting them. For example, this survey did not ask about smartphone ownership or operating systems used (eg, Android, Apple), cell phone service providers, utilization of different types of apps, or desire/need for mobile products for managing children’s chronic conditions. Furthermore this was a convenience sample that may not be representative of URM parents who do not seek primary care for their children (ie, rely on emergency departments) and does not reflect the usage and preferences of non-English speakers. Future studies of mobile technology use in pediatric settings should measure the usage of patients themselves (ie, children) and assess eHealth literacy to understand the capacity for content uptake by patients/caregivers.

Overall, this study confirmed that African American parents, even those with lower incomes, are frequent users of various mobile technologies. Although few are currently using such modalities for managing their children’s health, there was pervasive interest in doing so. Designers and researchers should note that mobile use varies by parent education and income and may need to account for service interruptions. Nonetheless, African American parents’ access and interest in using mHealth make it a promising platform for reducing pediatric health disparities.

## Abbreviations

GED: graduate equivalency degree
mHealth: mobile health
URM: underrepresented minority
